# Parental Alienation Syndrome (PAS). Psychological and legal implications

**DOI:** 10.1192/j.eurpsy.2023.1530

**Published:** 2023-07-19

**Authors:** M. Arrieta Pey, S. Rubio Corgo, A. Álvarez Astorga, A. M. Delgado Campos, C. Díaz Gordillo, A. C. Castro Ibáñez, M. Á. Álvarez de Mon González

**Affiliations:** 1 Psiquiatria Infanto-Juvenil; 2 Psiquiatria, Hospital Universitario Infanta Leonor, Madrid, Spain

## Abstract

**Introduction:**

The first definition of PAS, enunciated by Richard Gardner in 1985, refers to a disorder originating in the context of legal conflicts related to child custody. Its main characteristic would be a smear campaign by the child towards a parent, in the absence of plausible arguments. In this context, the child would experience an oppositional and dichotomous feeling towards his or her parents. In recent years, the presence of PAS has become increasingly important, both in the legal and health fields, largely due to the controversy and debate surrounding its approval and recognition, and there is currently no consensus on the matter.

**Objectives:**

The main objective of this work is to examine the current state of PAS in depth in the different fields in which it is emerging: the medical-scientific and legal spheres. The current controversies and debate, both scientific and legal, will be developed. Research will be carried out on the origin of the concept and its evolution, its symptomatic presentation, the neuropsychological consequences in minors, the role and legal value of expert reports, as well as the existing evaluation methods for the assessment of PAS.

**Methods:**

An extensive literature review was carried out on the subject in question, extracting information mainly from scientific articles, but also from legislative documents, manuals and books.

**Results:**

There are currently no specific laws regulating PAS in European countries. According to Article 10.2 of the Spanish Constitution, norms related to fundamental rights shall be interpreted according to the Universal Declaration of Human Rights. As a direct consequence of the chronic psychological stresses experienced by children, adaptive disorders may appear, often characterised by symptoms of anxiety and depression. In addition, a multitude of neuropsychological consequences have been observed not only in the affected child, but also in the adult he or she will become.

**Conclusions:**

Currently, there is a fervent debate about the validity and recognition of PAS as a diagnostic entity, spanning different disciplines, ranging from health to social and legal. In Europe, professionals in the scientific field have not reached an agreement regarding the approval of PAS. On the one hand, there are those for whom PAS is a verified phenomenon; on the other hand, there are those who flatly reject the existence of this phenomenon. The latter consider PAS an unscientific construct, referring to it as “court syndrome” or “patriarchal alienation syndrome”.

**Disclosure of Interest:**

None Declared

